# Alcohol and Sexual Assault

**Published:** 2001

**Authors:** Antonia Abbey, Tina Zawacki, Philip O. Buck, A. Monique Clinton, Pam McAuslan

**Affiliations:** Antonia Abbey, Ph.D., is an associate professor in the Department of Community Medicine, Wayne State University, Detroit, Michigan. Tina Zawacki, M.A., Philip O. Buck, M.A., and A. Monique Clinton, M.A., are research assistants in the Department of Community Medicine and doctoral students in the Department of Psychology, Wayne State University, Detroit, Michigan. Pam McAuslan, Ph.D., is an assistant professor in the Department of Behavioral Sciences, University of Michigan-Dearborn, Dearborn, Michigan

**Keywords:** sexual offense, assault and battery, aggressive behavior, AODR (alcohol or other drug [AOD] related) behavioral problem, AODR violence, AODR interpersonal and societal problems, personality trait related to social interaction, AOD expectancies, victim of abuse, social context, alcohol cue

## Abstract

Conservative estimates of sexual assault prevalence suggest that 25 percent of American women have experienced sexual assault, including rape. Approximately one-half of those cases involve alcohol consumption by the perpetrator, victim, or both. Alcohol contributes to sexual assault through multiple pathways, often exacerbating existing risk factors. Beliefs about alcohol’s effects on sexual and aggressive behavior, stereotypes about drinking women, and alcohol’s effects on cognitive and motor skills contribute to alcohol-involved sexual assault. Despite advances in researchers’ understanding of the relationships between alcohol consumption and sexual assault, many questions still need to be addressed in future studies.

Sexual assault^1^ of adolescent and adult women has been called a silent epidemic, because it occurs at high rates yet is rarely reported to the authorities (Koss 1988). Several reasons contribute to the underreporting of sexual assault cases. Many victims do not tell others about the assault, because they fear that they will not be believed or will be derogated, which, according to research findings, is a valid concern (Abbey et al. 1996*b*). Other victims may not realize that they have actually experienced legally defined rape or sexual assault, because the incident does not fit the prototypic scenario of “stranger rape.” For example, in a study by Abbey and colleagues (1996*b*), a woman wrote, “For years I believed it was my fault for being too drunk. I never called it ‘rape’ until much more recently, even though I repeatedly told him ‘no’.”

This article summarizes current knowledge about alcohol’s role in sexual assault and discusses questions that remain to be answered by future research. Alcohol’s contribution to sexual assault cannot be discussed without also describing the general characteristics of sexual assault; thus, this article alternates between providing information about sexual assault in general and contrasting this information with findings regarding alcohol-involved sexual assaults.

## The Prevalence of Sexual Assault and Alcohol-Involved Sexual Assault

The prevalence of sexual assault, both involving and not involving alcohol use, cannot be accurately determined, because it is usually unreported. Estimates of sexual assault prevalence have been based on a variety of sources, including police reports, national random samples of crime victims, interviews with incarcerated rapists, interviews with victims who seek hospital treatment, general population surveys of women, and surveys of male and female college students (Crowell and Burgess 1996). In such studies, the estimates’ adequacy varies with the sources of information used. Most researchers agree that the most reliable estimates derive from studies using multi-item scales—that is, measures containing several questions describing behaviors which constitute sexual assault in simple, nonlegal language (Koss 1988).

Based on such measures, conservative estimates suggest that at least 25 percent of American women have been sexually assaulted in adolescence or adulthood and that 18 percent have been raped. Furthermore, at least 20 percent of American men report having perpetrated sexual assault and 5 percent report having committed rape (Crowell and Burgess 1996; Spitzberg 1999; Tjaden and Thoennes 2000). Due to their accessibility, college student surveys tend to employ the most thorough measures of sexual assault by including the largest number of behaviorally specific questions. These studies suggest that approximately 50 percent of college women have been sexually assaulted, and 27 percent have experienced rape or attempted rape; in contrast, 25 percent of college men have committed sexual assault, and 8 percent have committed rape or attempted rape (Crowell and Burgess 1996; Koss 1988; Spitzberg 1999).

At least one-half of all violent crimes involve alcohol consumption by the perpetrator, the victim, or both (Collins and Messerschmidt 1993). Sexual assault fits this pattern. Thus, across the disparate populations studied, researchers consistently have found that approximately one-half of all sexual assaults are committed by men who have been drinking alcohol. Depending on the sample studied and the measures used, the estimates for alcohol use among perpetrators have ranged from 34 to 74 percent (Abbey et al. 1994; Crowell and Burgess 1996). Similarly, approximately one-half of all sexual assault victims report that they were drinking alcohol at the time of the assault, with estimates ranging from 30 to 79 percent (Abbey et al. 1994; Crowell and Burgess 1996). It is important to emphasize, however, that although a woman’s alcohol consumption may place her at increased risk of sexual assault, she is in no way responsible for the assault. The perpetrators are legally and morally responsible for their behavior.

Finally, alcohol consumption by perpetrators and victims tends to co-occur— that is, when one of them is drinking, the other one is generally drinking as well (Abbey et al. 1998; Harrington and Leitenberg 1994). Rarely is only the victim drinking alcohol. This finding is not surprising, because in social situations (e.g., in bars or at parties), drinking tends to be a shared activity. However, this finding complicates researchers’ efforts to disentangle the unique effects of alcohol consumption on the perpetrators’ versus the victims’ behavior.

## Common Characteristics of Non-Alcohol-Involved and Alcohol-Involved Sexual Assaults

Sexual assault occurs most commonly among women in late adolescence and early adulthood, although infants, as well as women in their 80s, have been raped (Crowell and Burgess 1996). Most sexual assaults that are reported to the police occur between strangers. These assaults, however, represent only a small proportion of all sexual assaults. At least 80 percent of sexual assaults occur among persons who know each other (Crowell and Burgess 1996).

Several studies in various populations have attempted to identify “typical” characteristics of sexual assault. Among college students, a typical sexual assault occurs on a date, at either the man’s or the woman’s home, and is preceded by consensual kissing. In addition, the assault involves a single assailant who uses no weapon, but twists the woman’s arm or holds her down. The woman, who believes that she has clearly emphasized her nonconsent, tries to resist through reasoning and by physically struggling (Koss 1988).

In a representative community sample, the typical sexual assault scenario involved a woman who was assaulted by a single assailant who was either an acquaintance or a friend and who used both verbal and physical pressure, which the woman tried to resist (Sorenson et al. 1987).

Although alcohol-involved and non-alcohol-involved sexual assaults share many characteristics, some differences exist. For example, sexual assaults involving alcohol consumption are more likely than other sexual assaults to occur between men and women who do not know each other well (e.g., strangers, acquaintances, or casual dates as opposed to steady dates or spouses). Furthermore, alcohol-involved sexual assaults tend to occur at parties or in bars, rather than in either person’s home (Abbey et al. 1996*a*).

## Investigating the Relationship Between Alcohol Consumption and Sexual Assault

Although alcohol consumption and sexual assault frequently co-occur, this phenomenon does not prove that alcohol use causes sexual assault. Thus, in some cases, the desire to commit a sexual assault may actually cause alcohol consumption (e.g., when a man drinks alcohol before committing a sexual assault in order to justify his behavior). Moreover, certain factors may lead to both alcohol consumption and sexual assault. For example, some fraternities encourage both heavy drinking and sexual exploitation of women (Abbey et al. 1996*b*). In fact, many pathways can prompt a man to commit sexual assault, and not all perpetrators are motivated by the same factors (Seto and Barbaree 1997). This article, therefore, describes several different ways in which alcohol consumption by the perpetrator and the victim can encourage sexual assault.

### Methods for Investigating Alcohol’s Role in Sexual Assault

Researchers have used two main approaches to examine alcohol’s role in sexual assault: (1) surveys of victims and perpetrators of sexual assault and (2) laboratory studies that examine alcohol’s effects on human behavior.

Each approach has its strengths and limitations.

Sexual assault is a particularly private, personal crime, and it is impossible for researchers to observe or fully simulate sexual assault. Thus, interviews with victims and perpetrators serve as the primary source of information regarding the circumstances under which the sexual assault occurred. Even the best-constructed surveys, however, have some limitations. When studies are conducted years after the sexual assault occurred, a person’s recall may be inaccurate, especially when the person was intoxicated at the time of the assault. Moreover, some participants may provide a somewhat distorted account of the events in order to avoid personal embarrassment. Finally, the surveys conducted to date vary in quality (e.g., sample size and validity of measures). This article focuses on only the findings of surveys that used large, representative samples and measures with established reliability and validity.

Laboratory studies are investigations in which participants consume either an alcoholic or a nonalcoholic beverage before their sexual or aggressive behavior is measured. The primary strength of this methodology is that it allows researchers to establish cause and effect for a certain behavior, because the participants are randomly assigned to the alcohol or nonalcohol condition. The major disadvantage of these studies is that for obvious ethical reasons, researchers cannot study directly the variable of interest (i.e., sexual assault). Instead, they must use proxy measures that may not accurately represent sexual assault experiences. For example, some investigators have used the participants’ responses to pornography as a proxy for sexual assault. Other researchers have asked participants to read and respond to stories about sexual assault. Although it is important to understand how people react to sexual assault victims and perpetrators, responses to a story may not reflect how people would behave if actually in a sexual assault situation.

In summary, surveys of victims and perpetrators cannot unequivocally demonstrate a cause-effect relationship between alcohol consumption and sexual assault, whereas laboratory studies cannot measure actual responses to sexual assault. Consequently, researchers must conduct both types of studies. Similar results obtained with both approaches increase confidence in the studies’ conclusions. The explanations of alcohol’s role in sexual assault reviewed in the following section have been examined in studies using such complementary methodologies. Much more research on this topic is needed, however, and specific suggestions for future research are presented at the end of the article.

## Pathways Through Which Alcohol Contributes to Sexual Assault

Theoretical explanations of sexual assault and of alcohol’s role in sexual assault consider both distal and proximal influences. Distal factors are influences that are temporally far removed from the assault; in contrast, proximal factors are influences that are temporally close to the assault. Distal predictors of sexual assault include personality characteristics, attitudes, and general life experiences of both the perpetrator and the victim. When examining alcohol as a distal factor, researchers focus on the relationship between the perpetrator’s and the victim’s long-term alcohol consumption patterns (e.g., regular heavy drinking) and sexual assault history as well as on beliefs about alcohol’s effects (i.e., expectancies) that may encourage alcohol-involved sexual assault. Proximal models of sexual assault focus on characteristics of the specific situations in which sexual assault occurs, such as whether alcohol consumption occurs, whether the setting is in an isolated area, and what the relationship is between the perpetrator and the victim. This section discusses both of these approaches (also see table).

### Perpetrators’ Personality Characteristics, Attitudes, and Experiences

Several studies that compared the characteristics of men who had committed sexual assault with those who had not noted the following differences (Seto and Barbaree 1997):

- With respect to personality traits, men who had committed sexual assault were more hostile toward women and lower in empathy compared with other men.
- With respect to attitudes, men who had committed sexual assault were more likely than other men to endorse traditional stereotypes about gender roles—for example, that men are responsible for initiating sex and women are responsible for setting the limits. Perpetrators of sexual assault also were more likely to endorse statements that have been used to justify rape—for example, “women say ‘no’ when they mean ‘yes’” and “women enjoy forced sex.” Finally, men who had committed sexual assaults were more likely to hold adversarial beliefs about relationships between men and women (e.g., “all’s fair in love and war”) and to consider the use of force in interpersonal relationships acceptable.
- With respect to their personal experiences, sexual assaulters were more likely than other men to have experienced abuse or violence as a child, to have been delinquent in adolescence, to have peers who viewed forced sex as acceptable, and to have had early and frequent dating and sexual experiences.

Heavy alcohol consumption also has been linked to sexual assault perpetration. In studies involving two different subject groups (i.e., incarcerated rapists and college students), men who reported that they drank heavily^2^ were more likely than other men to report having committed sexual assault (Abbey et al. 1994; Koss and Dinero 1988). General alcohol consumption could be related to sexual assault through multiple pathways. First, men who often drink heavily also likely do so in social situations that frequently lead to sexual assault (e.g., on a casual or spontaneous date at a party or bar). Second, heavy drinkers may routinely use intoxication as an excuse for engaging in socially unacceptable behavior, including sexual assault (Abbey et al. 1996*b*). Third, certain personality characteristics (e.g., impulsivity and antisocial behavior) may increase men’s propensity both to drink heavily and to commit sexual assault (Seto and Barbaree 1997).

Certain alcohol expectancies have also been linked to sexual assault. For example, alcohol is commonly viewed as an aphrodisiac that increases sexual desire and capacity (Crowe and George 1989). Many men expect to feel more powerful, disinhibited, and aggressive after drinking alcohol. To assess the influence of such expectancies on perceptions of sexual behavior, Norris and Kerr (1993) asked sober college men to read a story about a man forcing a date to have sex. Study participants reported that they would be more likely to behave like the man in the story when they were drunk, rather than when they were sober, suggesting that they could imagine forcing sex when intoxicated. Furthermore, college men who had perpetrated sexual assault when intoxicated expected alcohol to increase male and female sexuality more than did college men who perpetrated sexual assault when sober (Abbey et al. 1996*b*). Men with these expectancies may feel more comfortable forcing sex when they are drinking, because they can later justify to themselves that the alcohol made them act accordingly (Kanin 1984).

Attitudes about women’s alcohol consumption also influence a perpetrator’s actions and may be used to excuse sexual assaults of intoxicated women. Despite the liberalization of gender roles during the past few decades, most people do not readily approve of alcohol consumption and sexual behavior among women, yet view these same behaviors among men with far more leniency (Norris 1994). Thus, women who drink alcohol are frequently perceived as being more sexually available and promiscuous compared with women who do not drink (Abbey et al. 1996*b*). Sexually assaultive men often describe women who drink in bars as “loose,” immoral women who are appropriate targets for sexual aggression (Kanin 1984; Scully 1991). In fact, date rapists frequently report intentionally getting the woman drunk in order to have sexual intercourse with her (Abbey et al. 1996*b*).

### Victims’ Personality Characteristics, Attitudes, and Experiences

Parallel to research on perpetrators, numerous studies have compared the personality characteristics, attitudes, and life experiences of women who were sexually assaulted with those of other women. Overall, those analyses found only few significant effects and explain only small amounts of variance, indicating that women’s personal characteristics are not strong predictors of victimization.

Some differences exist, however, among women who have been victims of sexual assault and those who have not. Women who have been sexually assaulted are more likely than are other women to have experienced childhood sexual abuse, to have frequent sexual relationships, and to be heavy drinkers (Abbey et al. 1996*a*; Koss and Dinero 1989). Explanations of these findings focus on the long-term effects of childhood victimization (Wilsnack et al. 1997). Some victims of childhood sexual abuse cope with the resulting stress and negative emotions through early and frequent sexual relations and heavy drinking. These women may also be more likely to drink alcohol in potential sexual situations as a means of coping with their ambivalent feelings about sex. In turn, drinking in potential sexual situations increases women’s risk of being sexually assaulted, both because sexually assaultive men may view them as easy targets and because the women may be less able to resist effectively.

### Situational Factors

Sexual assault involves both sexual behavior and aggression; accordingly, researchers must consider situational influences (i.e., cues) relevant to both behaviors, such as the location or social situation in which the assault occurs. These cues may differ somewhat depending on the type of sexual assault (i.e., stranger sexual assault versus date sexual assault). In the case of sexual assaults that occur among strangers or people who have just met, men who drink heavily may frequent settings, such as bars and parties, where women also tend to drink heavily and where a man can easily find an intoxicated woman to target for a possible sexual assault. In these situations, alcohol may give men the “liquid courage” required to act on their desires and may reinforce their stereotypes about drinking women. For example, an incarcerated rapist interviewed by Scully (1991) stated that, “Straight, I don’t have the guts to rape. I could fight a man but not that.” (p. 124)

Alcohol consumption is also used by date rapists to excuse their behavior. For example, 62 percent of the college date rapists interviewed by Kanin (1984) felt that they had committed rape because of their alcohol consumption. These rapists did not see themselves as “real criminals,” because real criminals used weapons to assault strangers. In fact, some men may purposely get drunk when they want to act sexually aggressive, knowing that intoxication will provide them with an excuse for their socially inappropriate behavior.

As described earlier, at least 80 percent of all sexual assaults occur during social interaction, typically on a date. The fact that sexual assault often happens in situations in which consensual sex is a possible outcome means that a man’s interpretation of the situation can influence his responses. Consequently, additional situational factors are relevant to these types of sexual assaults. For example, American men are socialized to be the initiators of sexual interactions. Consequently, if a man is interested in having sex with a woman, he is likely to feel that he should make the first move. Initial sexual moves are usually subtle in order to reduce the embarrassment associated with potential rejection. Both men and women are used to this indirect form of establishing sexual interest and usually manage to make their intentions clear and save face if the other person is not interested (Abbey et al. 1996*b*). However, because the cues are subtle and sometimes vague, miscommunication can occur, particularly if communication skills are impaired by alcohol use.

As male-female interaction progresses, a woman who has been misperceived as being interested in sex may realize that her companion is reading more into her friendliness than she intended. However, she may not feel comfortable giving a direct message of sexual disinterest, because traditional female gender roles emphasize the importance of being nice and “letting men down easy.” The man, in turn, may not take an indirect approach to expressing sexual disinterest seriously. Research on the power of stereotypes, expectancies, and self-fulfilling prophecies demonstrate that when people have an expectation about a situation or another person, they tend to observe and recall primarily the cues that fit their hypothesis and to minimize or ignore the cues that contradict their hypothesis. Consequently, when a man hopes that a woman is interested in having sex with him, he will pay most attention to the cues that fit his expectation and disregard cues that do not support his expectation. Studies with both perpetrators and victims have confirmed that the man’s misperception of the woman’s degree of sexual interest is a significant predictor of sexual assault (Abbey et al. 1996*a*, 1998).

The process just described can occur even in the absence of alcohol use. However, alcohol consumption can exacerbate the likelihood of misperception, thereby increasing the chances of sexual assault. Before describing these dynamics, the laboratory research findings on alcohol’s effects on aggressive and sexual behavior should be reviewed.

### General Research on Alcohol’s Effects on Aggressive and Sexual Behavior

To determine which alcohol effects are attributable to alcohol’s pharmacology and which are attributable to culturally learned beliefs, researchers have utilized the balanced placebo design or some of its recent modifications (Martin and Sayette 1993; Rohsenow and Marlatt 1981). In the standard balanced placebo study, participants are randomly assigned to one of the following four groups:

- Participants who expect and receive an alcoholic beverage
- Participants who expect an alcoholic beverage but receive a nonalcoholic beverage
- Participants who expect a nonalcoholic beverage but receive an alcoholic beverage
- Participants who expect and receive a nonalcoholic beverage.

With this experimental design, effects that occur only in participants who received an alcoholic beverage, whether or not they expected it, can be considered to result from alcohol’s pharmacological actions. Conversely, effects that occur only in participants who expect to receive alcohol, whether or not they actually consume an alcoholic beverage, can be considered to result from alcohol expectancies.

Researchers who have examined the pharmacological versus psychological effects of alcohol have come to different conclusions depending on whether the variable of interest in the outcome was aggression or sexuality. The effects of alcohol on aggression appear to be principally pharmacological. Thus, in studies using the balanced placebo design, alcohol’s effects were usually observed in the participants who consumed alcohol, but not in the participants who only expected to consume alcohol (Ito et al. 1996). In addition, aggressiveness increased with the alcohol dose (Taylor and Chermack 1993).

Most investigators agree that alcohol’s effects on aggressive behavior are mediated by alcohol-induced cognitive deficits. Alcohol consumption disrupts higher order cognitive processes—including abstraction, conceptualization, planning, and problem-solving—making it difficult for the drinker to interpret complex stimuli. Thus, when under the influence of alcohol, people have a narrower perceptual field and can attend only to the most obvious (i.e., salient) cues in a given situation (Taylor and Chermack 1993). In aggression-inducing situations, the cues that usually inhibit aggressive behavior (e.g., concerns about future consequences or a sense of morality) are typically less salient than feelings of anger and frustration. Therefore, when a person is intoxicated, inhibitory cues are ignored or minimized, making aggression seem like the most reasonable response.

In contrast, studies of alcohol’s influence on sexual behavior have found more psychological effects. In men, high alcohol doses generally reduce physiological sexual responding, whereas low and moderate alcohol doses increase subjective sexual arousal. Many studies have demonstrated that men who believe they have consumed alcohol experience greater physiological and subjective sexual arousal in response to erotic materials depicting consensual and forced sex than do men who believe they have consumed a non-alcoholic beverage, regardless of what they actually drank (Crowe and George 1989).

Fewer studies have examined alcohol’s effects on sexual behavior in women, and the results have been inconsistent. This finding is generally explained in terms of society’s negative messages regarding women’s alcohol consumption and sexuality (Norris 1994). Thus, sexual behavior and drunken excess are considered less acceptable in women than in men, and unlike men, women must be concerned about being labeled as loose, or promiscuous. In addition, women are concerned about their increased vulnerability to sexual and nonsexual aggression when intoxicated. Consequently, women’s expectancies about alcohol’s sexual effects are less positive than men’s expectancies, because the social costs associated with alcohol use and sexual behavior are greater for women.

In summary, research suggests that alcohol exerts its effects on aggressive behavior principally through its pharmacological effects on cognitive processing, whereas alcohol’s effects on sexual behavior occur through pharmacological processes as well as psychological expectancies. Crowe and George (1989) summarized the literature by arguing that expectancies reduce “inhibitory conflict, enabling alcohol-induced cognitive impairments to disinhibit behavior. As inebriation increases, therefore, inhibition is reduced both by expectancies and by increasing inability to process inhibitory cues.” (p. 383)

### Alcohol’s Effects in Sexual Assault Situations

Abbey and colleagues (1994, 1996*b*) have developed a model to explain the role of alcohol in sexual assaults perpetrated by acquaintances. The model suggests that alcohol acts at two distinct points during the interaction between the perpetrator and the victim to increase the likelihood of sexual assault. The first point is during the early stages of the interaction, when the man is evaluating the likelihood that his companion wants to have sex with him. This evaluation is an ongoing process. During a date or other social interaction, many points occur at which a man evaluates the potential sexual meaning of a female companion’s verbal or non-verbal cues. Alcohol can contribute to the misperception of the woman’s cues in such a way that the man perceives her as being more encouraging than she really is because of alcohol’s effects on his cognitive functioning. The woman experiences the same cognitive deficits as the man does if the woman also consumes alcohol. Thus, if she feels that she has made it clear that she is not interested in sex at this point, alcohol consumption will make her less likely to process the man’s cues indicating that he has misread her intentions.

This model is difficult to test directly, however, because researchers must rely on participants’ retrospective recall of sexual assault situations. Nevertheless, a study among college men found that increased alcohol consumption in social situations increased the participants’ misperceptions of women’s cues (Abbey et al. 1998). The extent of such misperceptions, in turn, was related to the frequency with which the men committed sexual assault. In a parallel study among college women, drinking in situations in which men misperceived the women’s sexual intentions increased the likelihood that the women became victims of a sexual assault (Abbey et al. 1996*a*). In addition, Testa and Livingston (1999) found that women who had been drinking prior to being sexually assaulted reported that their intoxication made them take risks that they normally would avoid. For example, the women felt comfortable accepting a ride home from a party with a man they did not know well or letting an intoxicated man into their apartment.

The second point at which alcohol plays a role in sexual assault is when the man forces sex on a woman against the woman’s wishes. Alcohol is not necessary in this scenario, because some men feel entitled to force sex on women if they feel that they have been “led on” or teased (Abbey et al. 1994). The cognitive deficits associated with alcohol consumption, however, can enhance a man’s likelihood of behaving aggressively, because an intoxicated man may have more difficulty generating non-aggressive solutions to gaining sexual satisfaction. Thus, when a man is intoxicated, he can more easily focus on his immediate sexual gratification, sense of entitlement, and anger, rather than on his internalized sense of appropriate behavior, future regret, the victim’s suffering, or the possibility that he will be punished for his actions. Furthermore, in laboratory studies, intoxicated men tend to retaliate strongly when they feel threatened, and once they begin behaving aggressively, they can only be stopped with great difficulty (Taylor and Chermack 1993). Accordingly, if an intoxicated man feels that his female companion has implicitly agreed to sex, he may perceive any resistance as a threat and thus become aggressive in retaliation. The effect of his aggressive behavior is further exacerbated if the woman is intoxicated, because alcohol’s effects on motor skills may limit her ability to resist effectively (Koss and Dinero 1989).

To support the aforementioned hypotheses, researchers must demonstrate that sexual assaults involving intoxicated perpetrators and/or intoxicated victims are more likely than other sexual assaults to include extreme levels of forced sex, more violent behavior, and more injuries to the victim. In fact, some studies indicate that completed rapes (as opposed to attempted rapes) are more common among intoxicated victims than among sober victims, suggesting that intoxicated women are less able than sober women to resist an assault effectively (Abbey et al. 1996*b*; Harrington and Leitenberg 1994).

Surprisingly, recent studies focusing on alcohol consumption among men have not confirmed that drinking men are more likely to successfully commit rape, as opposed to attempt rape but fail in their efforts. Nonetheless, more serious victim injuries have been associated with alcohol consumption by the perpetrator (Martin and Bachman 1998; Ullman et al. 1999). These studies, however, suffer from serious methodological limitations with respect to measuring the perpetrators’ alcohol consumption. The investigators assessed only whether the perpetrators consumed any alcohol before the assault and did not determine how much alcohol the perpetrators consumed or how far in advance of the assaults the drinking occurred. Consequently, the studies did not provide conclusions as to how intoxicated the perpetrators were at the times of the assaults. For example, men who had consumed only one drink several hours before the assault may not have been intoxicated at all. Conversely, men who were extremely intoxicated may have experienced sexual and motor impairments that made sexual assault completion unlikely.

## Suggestions for Future Research

As noted earlier in this article, investigators have used two distinct research methodologies to gather information about alcohol’s role in sexual assault: (1) surveys of victims and perpetrators and (2) laboratory studies. Further research using both methodologies would enable investigators to describe more accurately the characteristics of alcohol-involved sexual assault and to test potential causal mechanisms.

Several limitations exist in current surveys of sexual assault. First, most researchers only collect data at one point in time, making it impossible to distinguish which beliefs or experiences came first, those relating to alcohol use or those relating to sexual experiences. For example, Wilsnack and colleagues (1997) found that heavy drinking can be both an antecedent and a consequence of sexual assault. Long-term prospective studies that follow the same group of people for several years are needed to determine whether heavy drinking precedes the sexual assault or vice versa. Similarly, such prospective studies must measure other alcohol-related factors (e.g., alcohol expectancies and usual alcohol consumption in social situations) before and after a sexual assault occurs in order to demonstrate whether these factors are causes or consequences of sexual assault.

Second, sexual assault researchers and alcohol researchers must interact more closely to identify causes contributing to alcohol-involved sexual assault. For example, sexual assault researchers have identified numerous personality and attitudinal variables that predict sexual assault perpetration (Crowell and Burgess 1996). Similarly, alcohol researchers have examined personality and situational variables related to sexual and aggressive behavior in the laboratory (Taylor and Chermack 1993). Additional research is needed, however, to determine the extent to which some of the concepts examined in laboratory studies of alcohol’s effects on aggression and sexuality help explain alcohol-involved sexual assault. For example, if intoxication encourages sexual assault through its effects on cognitive skills, do individual differences in general cognitive functioning relate to alcohol-involved sexual assault perpetration? Alternatively, impulsivity has been linked to both aggression and sexual assault; however, researchers have not yet investigated whether impulsive sexual assault perpetrators respond more aggressively when drinking.

Third, most sexual assault researchers are not well versed in the alcohol literature; accordingly, their measurements of alcohol consumption are often inadequate. For example, study participants often are asked only the simple dichotomous question, “Did you drink alcohol?” To better assess perpetrators’ and victims’ level of intoxication and the resulting impairment, investigators must ask additional questions about the number of drinks consumed, the time period in which they were consumed, the person’s normal drinking level and drinking pace, and the degree of subjective intoxication. Although no set of retrospective self-report items allows researchers to calculate a person’s blood alcohol concentration precisely, more detailed questions, such as the ones listed, will enable scientists to address more complex hypotheses about alcohol’s role in sexual assault, such as the following:

- Is the perpetrator’s level of intoxication linearly related to the level of force used or is the relationship more complex such that at the highest levels of intoxication, cognitive and motor skills are so impaired that the level of force declines?
- When a woman drinks more or faster than usual, does she increase her risk of being sexually assaulted?

Additionally, because in most alcohol-involved sexual assaults both the perpetrator and the victim drink, the independent influences of the perpetrator’s and the victim’s alcohol consumption are difficult to examine. At a minimum, researchers must acknowledge this problem (Martin and Bachman 1998). Ideally, investigators should conduct studies with large enough samples to allow analyses of the separate effects of perpetrators’ and victims’ alcohol consumption.

Fourth, qualitative research will enable researchers to understand more fully the mechanisms through which alcohol contributes to sexual assault—for example, by addressing the following types of questions:

- How often do men select a woman as a target because she has been drinking, and what strategies do the men use to isolate and control her?
- Does alcohol’s role differ in sexual assaults among strangers, acquaintances, casual dates, and steady dating partners?
- When a man is drinking alcohol, does he miss cues indicating that the woman is not interested in sex or does he simply not care about her feelings?
- What is the role of peer pressure in encouraging men both to drink heavily and to force sex?
- What types of warning signs occur (and which of those signs do women tend to observe) before a sexual assault, and do intoxicated women notice fewer cues or interpret them as less threatening than do sober women?
- What types of environmental factors encourage alcohol-induced sexual assault? Is it more common at certain types of bars or parties?

Detailed interviews with victims and perpetrators from different ethnic and cultural backgrounds are needed to better understand which pathways are most common under what circumstances.

Finally, because even the best-constructed prospective interview study allows for alternative causal explanations, further laboratory research also is needed. In such studies, researchers can randomly assign participants to groups receiving alcoholic or nonalcoholic beverages, thereby insuring that differences in the participants’ behavior result from alcohol consumption, rather than from other factors, such as personality characteristics or environmental circumstances. A major challenge is to develop reasonable yet ethical proxies for sexual assault that can be used in the laboratory. Furthermore, most laboratory studies currently conducted on alcohol include only men. Consequently, more laboratory research with female participants is needed to increase understanding of alcohol’s effects on women’s sexual perceptions and behavior and to allow for direct comparisons of men’s and women’s responses. Such studies do not always need to simulate sexual assault to inform theory about it. Laboratory research that examines the processes through which alcohol exacerbates miscommunication between women and men and influences the cognitive and affective responses of women and men to sexual disagreements can help guide prevention programs.

## Figures and Tables

**Table:** Summary of Explanations for Alcohol-Related Sexual Assault, Including Distal Factors, Which Are Temporarily Removed From the Assault, and Personal Factors, Which Are Temporarily Close to the Assault

	Perpetrators	Victims
**Distal Factors**	General, heavy alcohol consumption	General, heavy alcohol consumption
	Alcohol expectancies about sex, aggression, and disinhibition	Childhood sexual abuse
	Stereotypes about drinking women being sexually available and appropriate targets	
**Situational Factors**	Heavy drinkers spend time in bars and at parties	Heavy drinkers spend time in bars and at parties
	Drinking is used as an excuse for socially unacceptable behavior	Alcohol’s cognitive impairments reduce ability to evaluate risk
	Alcohol’s cognitive impairments enhance misperception of the woman’s friendly cues as sexual	Alcohol’s motor impairments reduce ability to resist effectively
	Alcohol’s cognitive impairments facilitate an aggressive response if the man feels he has been “led on”	

## Footnotes

1 For a definition of this and other terms used in this article, see the glossary, p. 50.

2 The term “heavy drinking” is defined differently by each researcher and therefore is used here as in the original articles cited.

## GLOSSARY

Acquaintance rape: *Rape* committed by someone that the victim knows, such as an acquaintance, friend, co-worker, date, or spouse. Most rapes are acquaintance rapes.
Alcohol expectancies: A person’s beliefs about the effects that alcohol consumption will have on himself or herself as well as on other people.
Alcohol expectancy set: The practice in laboratory research of telling participants that they have consumed alcohol, regardless of what the participants actually are given to drink.
Alcohol-involved rape: *Rape* in which the perpetrator, the victim, or both are under the influence of alcohol at the time of the incident.
Attempted rape: An act that fits the definition of *rape*, in terms of the strategies used, but does not result in penetration.
Childhood sexual abuse: Sexual abuse that occurs to a child (the term “child” is generally defined as age 13 or younger).
Date rape: *Rape* committed by someone that the victim is dating. Among college students, approximately one-half of all rapes are committed by a date.
Marital rape: *Rape* committed by the victim’s spouse. Marital rape often is committed in association with verbal and physical abuse.
Rape: A *sexual assault* involving some type of penetration (i.e., vaginal, oral, or anal) due to force or threat of force; lack of consent; or inability of the victim to provide consent due to age, intoxication, or mental status. Rape laws vary by State; however, the aforementioned description conforms to the definition used at the Federal level and by most States.
Sexual assault: The full range of forced sexual acts, including forced touching or kissing; verbally coerced intercourse; and vaginal, oral, and anal penetration. Researchers typically include in this category only acts of this nature that occur during adolescence or adulthood; in other words, *childhood sexual abuse* is defined separately. Both men and women can be sexually assaulted and can commit sexual assault. The vast majority of sexual assaults, however, involve male perpetrators and female victims.
Stranger rape: *Rape* committed by someone that the victim does not know. Less than 20 percent of rapes are committed by strangers, although most people believe that stranger rape is the prototypical rape.
